# Potential biomarkers for adult acute myeloid leukemia minimal residual disease assessment searched by serum peptidome profiling

**DOI:** 10.1186/1477-5956-11-39

**Published:** 2013-08-03

**Authors:** Ju Bai, Aili He, Wanggang Zhang, Chen Huang, Juan Yang, Yun Yang, Jianli Wang, Yang Zhang

**Affiliations:** 1 Department of Hematology, Second Affiliated Hospital, Medical School of Xi’an Jiaotong University, 157 Xiwu Road, Xincheng District, Xi’an, Shaanxi 710004, PR China; 2 Department of Genetics and Molecular Biology, Medical school of Xi’an Jiaotong University/Key Laboratory of Environment and Disease-Related Gene, Ministry of Education, 76 Western Yanta Road, Xi’an, Shaanxi 710061, PR China

**Keywords:** Weak cation exchange magnetic beads, MALDI-TOF-MS, Serum peptidome profiling, Adult acute myeloid leukemia, Minimal residual disease

## Abstract

**Background:**

Post treatment minimal residual disease (MRD) determination contributes to impending relapse prediction, chemotherapy response and clinical outcomes assessment, guiding clinicians to develop reasonable and effective individual chemotherapy options after induction/consolidation. This study was to identify serum candidate peptides for monitoring adult acute myeloid leukemia (AML) MRD.

**Results:**

47 statistically different expressed peptide peaks were obtained in the molecular weight range of 700-10000 Da. Quick classifier (QC) model had optimal distinction efficiency, in the training set with a sensitivity of 90% and a specificity of 93.33%. Peptides were identified as ubiquitin-like modifier activating enzyme 1(UBA1), isoform 1 of fibrinogen alpha chain precursor and platelet factor 4(PF4). The peptide up-regulated in newly diagnosed AML patients were decreased to the normal level after CR. When refractory & relapsed, relative intensity was elevated again. Results were contrary to down-regulated peptide peaks. Western blot demonstrated that levels of the UBA1 protein did not differ between the leukemia and normal cells. Levels of isoform 1 of fibrinogen alpha chain precursor protein and PF4 protein were both decreased in leukemia cells comparing with normal cells. The serum levels of the PF4 in the newly diagnosed AML patients and healthy controls were significantly different. Further correlation analysis did not indicate the correlated relation between platelet counts and PF4 content, the correlation coefficient was 0.097. Kaplan–Meier analyses of overall survival showed that relative intensity of peptides was correlated with patient’s clinical outcome.

**Conclusions:**

We speculate the peptides can be used as potential markers for monitoring minimal residual disease and clinical outcome assessment.

## Background

Acute myeloid leukemia(AML), a clonal expansion, accumulation and infiltration of myeloid hematopoietic blasts, is a highly heterogeneous hematological malignancy comprising many entities for which distinct treatment strategies are pursued [1]. Although M3 is a success story in AML oncology (cure rate more than 90%), clinical effects in non-M3 AMLs lag behind those in M3 [2]. The poor long-term disease-free survival rates of adult AML is mainly due to therapy-related mortality, failure of induction chemotherapy and early relapse [3].Risk stratification adapted therapies based on prognostic factors will help to improve the clinical outcomes. But indeed, patients with favorable karyotype accounts for approximately 40% to 50% of low-risk AML will eventually experience a relapse. For patients falling into the intermediate risk karyotype (~60%), post remission strategies-planning lacks accepted criteria. Lots of studies have shown that minimal residual disease (MRD) positive patients are at high risk of relapse, while MRD-negative cases manifest lower risk of relapse in patients with acute promyelocytic leukemia and acute lymphoblastic leukemia clinically. Nowadays, post treatment MRD determination appears to be appropriate in extrapolating the risk of relapse, assessing chemotherapy response and planning individual chemotherapy regimen after induction/consolidation [4].

Leukemia relapse is mainly due to the presence of MRD. Leukemia MRD level monitoring contributes to anticipation of impending relapse and assessment of clinical outcomes, guiding clinicians to develop reasonable and effective treatment options so that patients can avoid unnecessary chemical drug toxicity. Currently,MRD monitoring is mainly through detecting remaining leukemia cells in bone marrow by multi-parameter flow cytometry phenotype analysis and real-time quantitative polymerase chain reaction (RTQ-PCR). But multi-parameter flow cytometry phenotype analysis can’t be carried out in lots of hospitals and lack standardized procedure, the number of fusion genes known in acute leukemia is limited and RTQ-PCR assays shows poverty in standardized cut-offs. Moreover, bone marrow aspiration is invasive and increases the patient’s pain. Because most non-M3-AML patients lack specific fusion genes, so after every stage of chemotherapy, the response is mainly judged by whether the leukemia cells in the bone marrow are less than 5%. As bone marrow aspiration site is single, leukemia cells are not typical after multiple chemotherapy, bone marrow smears need long-term film-reading experience and skilled clinical hematological workers to read. Serum is easily accessible, can record different physiological or pathological conditions at any time and readily accepted by patients, therefore, it becomes one of the best sources for biomarkers researching.

Serum peptide profile method is known as “The new health fingerprint library” technology and has been accepted worldwide. It is through analysing and comparing differences in the expression of serum peptides between target population and normal healthy population, to find multiple different-expressed serum peptides, to map out disease-specific serum peptide spectrum, to diagnose disease, to clarify the possible pathogenesis and resistance mechanism, and to determine prognosis [5].

Proteomics technology has been applied to study hematological malignancies in some previous researches. The traditional two-dimensional gel electrophoresis (2-DE)-based separation technology combined matrix assisted laser desorption ionization time of flight mass spectrometry (MALDI-TOF-MS) or Protein chip-surface enhanced laser desorption ionization time of flight mass spectrometry (SELDI-TOF-MS) technology were mainly used to study cell lines and/or bone marrow cells. The aim of those researches was to find early diagnostic markers, to predict the prognosis of hematologic malignancies, to explore the molecular mechanisms of anti-cancer drugs and to develop molecular targeted drugs based on theses biomarkers. Although the cultured cell lines have high purity and are easy to operate, but they are difficult to reflect the real situation of the disease. Due to the complex composition and high cell heterogeneity of bone marrow, researchers analyzed serum samples of different hematologic malignancies in recent years using 2-DE combined with MALDI-TOF-MS or Protein chip-SELDI-TOF-MS, for instance, Albitar [6], Zou [7] and Mohamedali [8] had established the diagnostic model of leukemia with high sensitivity and specificity, and further identified the leukemia-related markers, such as Rho-GDP dissociation inhibitor autoantibodies, alpha enolase, aldolase enzyme A and so on [9]. These markers play an important role in the early diagnosis, differential diagnosis and pathogenesis of acute leukemia.

In order to facilitate peptide identification, we replaced solid chip with beads for sample purification and enrichment, and we employed MALDI-TOF MS technology for mass spectra acquisition, and highly sophisticated data mining algorithms for inspection and comparison of data sets as well as for the discovery of complex biomarker pattern models [10]. The ClinProt technology has lots of advantages, such as a large separation capacity [11], enriched samples easy to elute for further identification [12], simple and quick operation, high-throughput, software parallelization analysis, differentially expressed forms, etc. [13]. ClinProt technology-based serum peptide spectra was first used in ovarian cancer research [14], then widely used for early diagnosis, pathogenesis interpretation, therapeutic targets selection in melanoma [15], colorectal [16], breast cancer and other solid tumors [17,18]. Previously, our group applied ClinProt system to compare and analysis the difference between newly diagnosed multiple myeloma (MM) and healthy controls in serum peptide spectrum, and we established a diagnostic model with high sensitivity and specificity based on Supervised Neural Network Algorithm (SNN) [19].

In view of this, this study used weak cation exchange magnetic beads (MB-WCX) combined with MALDI-TOF-MS to analyze serum peptide profiling of adult AML patients in different disease groups (newly diagnosed group, complete remission (CR) group, refractory & relapsed group) and healthy control group, and then used high performance liquid chromatography electro spray ionization tandem mass spectrometry (HPLC-ESI-MS/MS) to identify candidate peptides. These protein fragments were validated by immunoblotting. Three peptides are identified as ubiquitin-like modifier activating enzyme 1(UBA1), isoform 1 of Fibrinogen alpha chain precursor and platelet factor 4(PF4). They are correlated with AML clinical outcome and would be appropriately adapted for predicting AML relapse, monitoring MRD and predicting prognosis in clinical practice.

## Results

### Repeatability of ClinProt system based peptidome detection

ClinProt system repeatability was evaluated based on the serum peptide fingerprints of the same serum sample processed in triplicate (Additional file 1: Figure S1). The two figures show that the peptide peaks are consistent in the three detections, indicating that ClinProt system has good repeatability.

Moreover, coefficient of variation (CV) value was used to evaluate repeatability and stability of serum peptide fingerprint in international. It is required that CV should be less than 30% [20]. In this study, the CV of the relative intensity of peptides in MALDI-TOF-MS peptide spectra was calculated to evaluate repeatability of the serum peptide fingerprints method (Table 1). The average CV value of within-run arrays was 14.7% (5.6%-24.8%) and that of between-run arrays was 15.6% (6.4%-27.3%).

**Table 1:** Reproducibility of mass spectra processed by ClinProt system

**m/z**	**Within-run arrays**		**Between-run arrays**	
	**MRI%**	**CV%**	**MRI%**	**CV%**
973.15	4.2	14.2	4.1	14.3
1866.09	8.8	9.8	8.6	10.5
2661.27	2.1	8.3	2.2	8.6
3443.92	4.9	12	5.2	12.4
4089.70	3.4	23	3.2	23.4
4208.76	13.1	16.5	13.6	17.2
5902.57	10.1	14.6	10.8	15.3
6628.07	7.2	18.1	6.9	20.9
7762.87	6.1	24.8	5.8	27.3
9288.31	1.7	5.6	1.6	6.4

*m/z* mass to charge ratio, *MRI* mass relative intensity, *CV* coefficient of variation.

### Analysis of newly diagnosed AML and healthy control serum peptide fingerprint by ClinProt system

**Figure 1:**
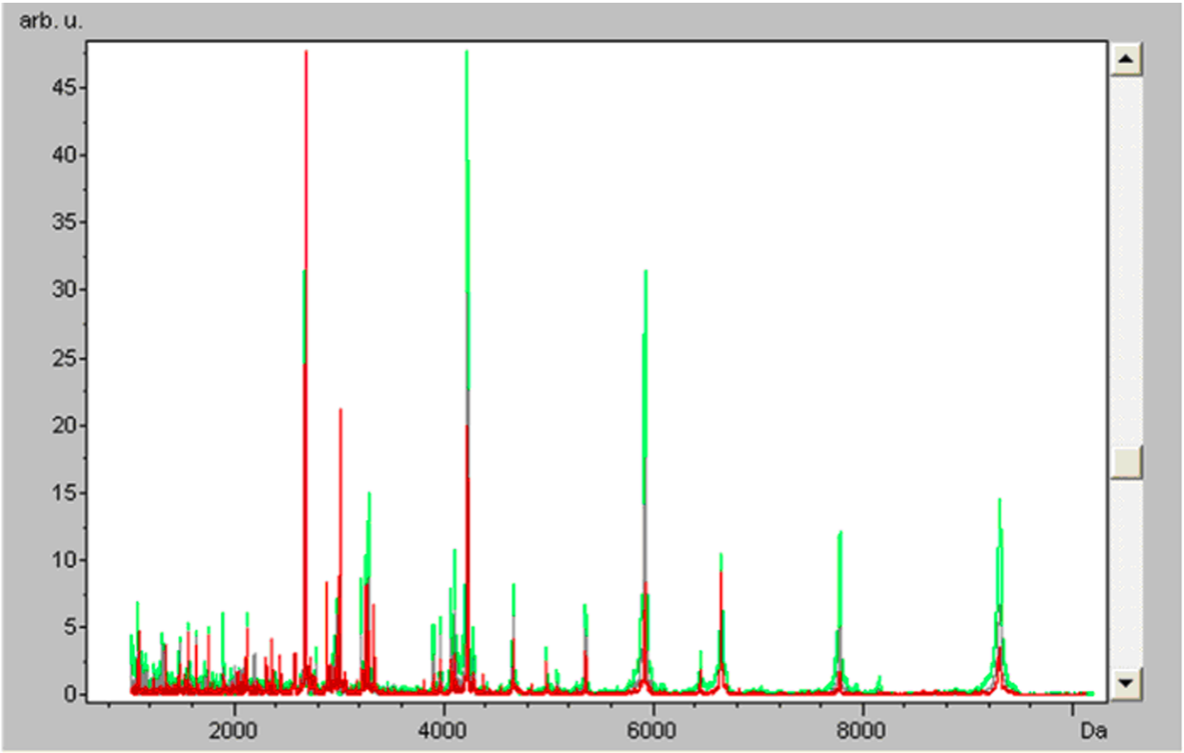
**Serum peptide fingerprint and cluster analysis of newly diagnosed AML and healthy control.** Comparison of serum peptide fingerprints between AML patients and healthy controls showed that peak number and intensity of the two groups were completely different. (Red: AML newly diagnosed group Green: Healthy control group).

To screen serum peptides of interest for AML, we made the comparison in serum peptide fingerprints between AML patients and healthy controls, showing that peak number and intensity of newly diagnosed AML patients’ serum peptide fingerprint were completely different from healthy controls’ (Figure 1), these significant different mass peaks were likely to be the AML potential characteristic peptides.

Comparative analysis of peptide fingerprint revealed that there were a total of 47 different expressed peptide peaks with statistical significance in the MW range of 700–10000 Da (p < 0.05). Among which, the expressions of 12 peptides were up-regulated and that of 35 peptides were down-regulated in AML newly diagnosed group (Table 2).

**Table 2:** Different expression peptides between newly diagnosed AML group and healthy control group

**Index**	**Mass**	**Dave**	**P value**	**Ave1**	**Ave2**	**StdDev1**	**StdDev2**	**Expression change**
48	4208.76	29.66	0.00000192	10.64	40.31	10.37	13.98	**↓**
12	1866.09	18.67	0.000489	22.08	3.41	25.9	1.6	**↑**
60	5902.57	16.49	0.0000421	10.02	26.51	9.35	11.59	**↓**
64	6628.07	9.4	0.00000388	13.85	4.45	7.71	3.24	**↑**
69	9286.53	7.84	0.00000192	2.34	10.19	2.03	4.38	**↓**
34	3262.18	7.49	0.0000723	5.78	13.27	7.05	6.55	**↓**
42	4089.7	7.39	0.00000108	2.69	10.08	1.39	4.03	**↓**
66	7762.87	6.42	0.00000108	1.94	8.36	1.25	3.35	**↓**
4	1282.99	5.83	0.00000203	2.57	8.41	0.63	6.16	**↓**
52	4643.21	5.66	0.0000168	2.37	8.03	1.75	4.4	**↓**
5	1299.56	5.53	0.00000108	2.33	7.87	0.57	5.26	**↓**
30	2990.42	5.3	0.00000146	6.88	1.57	5.08	0.68	**↑**
36	3315.58	5.05	0.00000203	7.05	2	3.74	1.15	**↑**
31	3191.17	4.61	0.00000625	2.7	7.31	1.63	3.51	**↓**
19	2168.67	4.45	0.0000573	2.04	6.5	0.71	5.51	**↓**
39	3950.86	4.17	0.00000203	2.56	6.73	1.27	2.76	**↓**
20	2184.85	4.05	0.0116	2.54	6.59	1.06	5.72	**↓**
29	2952.24	4.03	0.00616	4.57	8.6	3.14	5.22	**↓**
47	4192.99	4.01	0.00000515	2.46	6.48	1.31	2.79	**↓**
18	2105.44	3.74	0.0000127	3.05	6.79	1.55	2.99	**↓**
56	5335.01	3.21	0.000605	3.42	6.62	2.95	3.29	**↓**
40	4052.81	3.07	0.000206	3.13	6.21	2.83	2.37	**↓**
62	6430.27	3.02	0.0000335	4.67	1.66	3.69	1.13	**↑**
2	1100.81	2.86	0.000259	2.62	5.48	1.01	3.23	**↓**
13	1982.13	2.55	0.000137	2.21	4.75	0.63	3.1	**↓**
37	3882.23	2.53	0.000257	2.19	4.73	1.01	2.17	**↓**
16	2062.28	2.38	0.0000723	1.69	4.07	0.45	2.68	**↓**
25	2768.83	2.28	0.00000108	1.81	4.09	0.51	1.52	**↓**
15	2037.71	2.26	0.0075	1.84	4.1	0.35	3.12	**↓**
17	2069.96	2.19	0.000749	2.02	4.21	0.46	2.65	**↓**
59	5864.9	2.18	0.000247	2.47	4.65	1.15	1.85	**↓**
53	4818.02	2.17	< 0.000001	2.68	0.51	3.32	0.18	**↑**
50	4265.7	1.88	0.000749	2.46	4.34	1.07	1.85	**↓**
14	2021.95	1.8	0.00326	4.64	2.84	2.37	3.02	**↑**
28	2932	1.59	0.000605	2.63	4.22	0.81	1.56	**↓**
46	4167.87	1.56	0.0000437	1.69	3.25	0.84	1.02	**↓**
41	4071.18	1.39	0.000029	1.53	2.92	0.38	0.94	**↓**
33	3240.42	1.23	0.00958	7.47	8.7	9.41	3.17	**↓**
65	6662.81	1.13	0.0000187	2.28	1.15	0.93	0.56	**↑**
61	5957.41	1.08	0.0000299	1.06	2.14	0.26	0.95	**↓**
32	3216.57	1.04	0.00357	2.87	1.83	1.45	0.53	**↑**
63	6526.9	0.79	0.00000268	1.37	0.58	0.52	0.25	**↑**
44	4121.48	0.78	0.000259	1.62	2.4	0.38	0.71	**↓**
38	3933.89	0.77	0.0000406	1.42	2.19	0.38	0.65	**↓**
21	2561.11	0.69	0.0309	2.59	1.9	1.27	0.63	**↑**
43	4108.19	0.66	0.000357	1.37	2.03	0.42	0.56	**↓**
45	4152.3	0.18	0.0206	2.41	2.22	2.65	0.68	**↑**

*Index* peptide peak index, *Mass* mass to charge ratio value, *Dave* differences of average peak intensity between newly diagnosed AML group and healthy control group, *P value* p value of t-test, *Ave1* average peak intensity of newly diagnosed AML group, *Ave2* average peak intensity of healthy control group, *StdDev1* standard deviation of the peak intensity average of newly diagnosed AML group, *StdDev2* standard deviation of the peak intensity average of healthy control group.

### Establishment QC diagnostic model and blind verification

Genetic algorithm (GA), SNN and QC were applied to establish diagnostic model for distinguishing newly diagnosed AML from healthy control. Among them, QC model composed of three peptides had optimal distinction efficiency (90% sensitivity and 93.33% specificity) in the training set.

**Figure 2:**
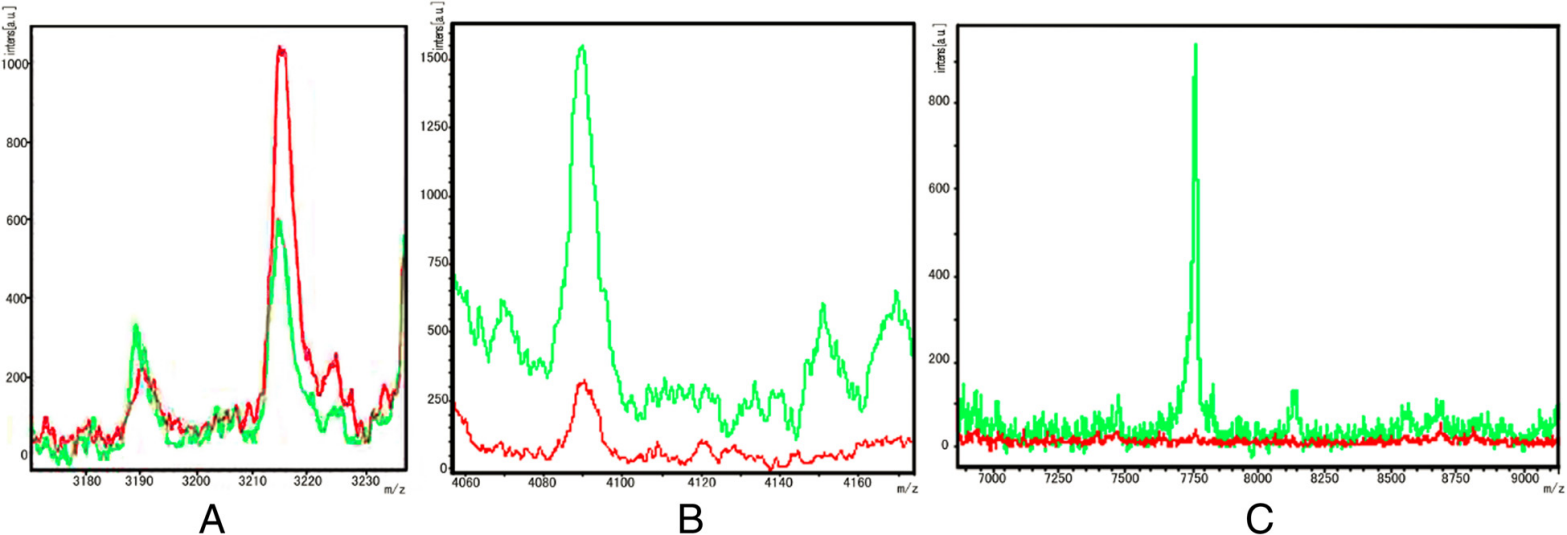
**Different relative intensities of three peptide peaks between AML newly diagnosed group and healthy control group. (A)** Upregulated of the peptide with MW of 3216.57 Da in AML newly diagnosed group. **(B)** Down regulated of the peptide with MW of 4089.7 Da in AML newly diagnosed group. **(C)** Down regulated of the peptide with MW of 7762.87 Da in AML newly diagnosed group. (Red: AML newly diagnosed group Green: Healthy control group).

In the QC model, the peptide with MW of 3216.57 Da was up-regulated in AML newly diagnosed group (Figure 2A), peptides with MW of 4089.7 Da and 7762.87 Da were both down-regulated (Figure 2B,C). Blind test verified that this model correctly identified 40 cases out of total 42 AML cases and 39 healthy cases from 42 healthy controls.

### Peptide identification

**Figure 3:**
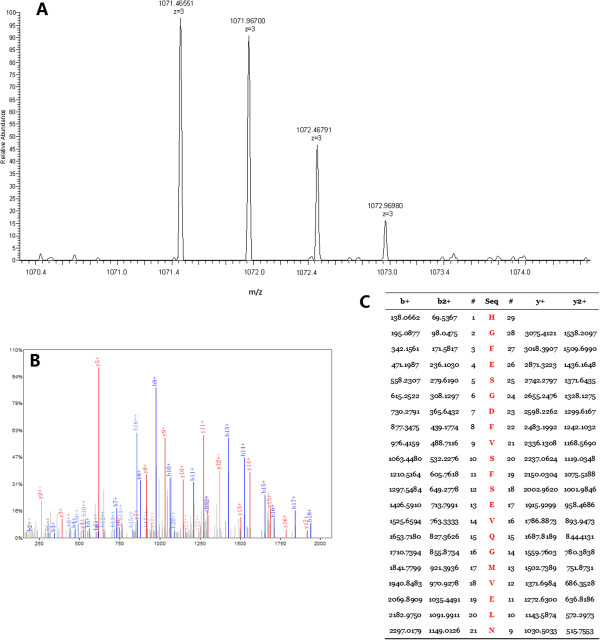
**MS/MS map of peptide with MW of 3216.57 Da. (A)** The enlarged picture of peptide with MW of 3216.57 Da. **(B)** The b and y ions spectra used to identify the peptide with MW of 3216.57 Da. **(C)** The sequence of the peptide with MW of 3216.57 Da.

**Figure 4:**
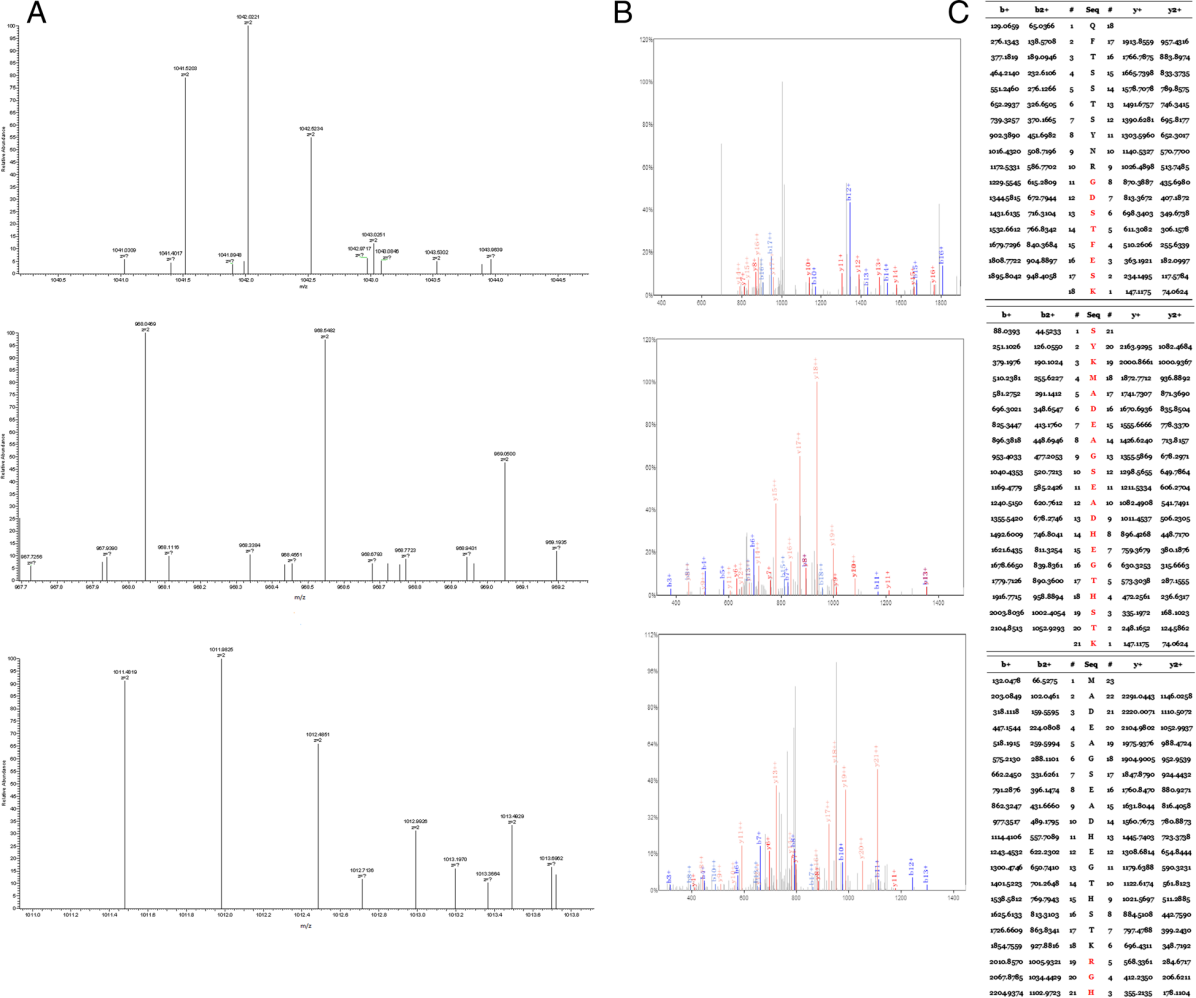
**MS/MS map of peptide with MW of 4089.7 Da. (A)** The enlarged picture of peptide with MW of 4089.7 Da. **(B)** The b and y ions spectra used to identify the peptide with MW of 4089.7 Da. **(C)** The sequence of the peptide with MW of 4089.7 Da.

**Figure 5:**
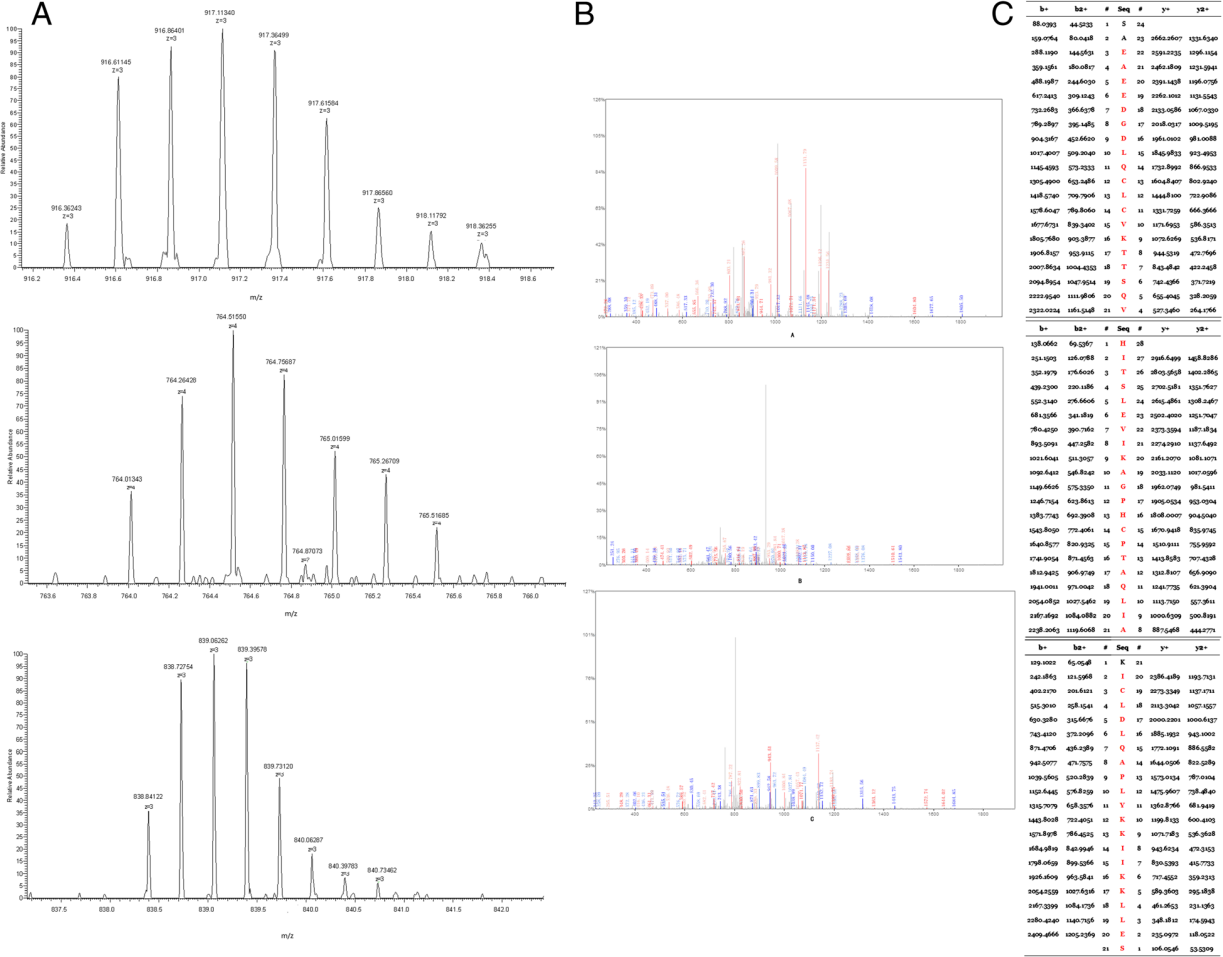
**MS/MS map of peptide with MW of 7762.87 Da. (A)** The enlarged picture of peptide with MW of 7762.87 Da. **(B)** The b and y ions spectra used to identify the peptide with MW of 7762.87 Da. **(C)** The sequence of the peptide with MW of 7762.87 Da.

The three peptides in QC model were purified and identified by HPLC-ESI-MS/MS. Data analysis software Bioworks Browser 3.3.1 SP1 was performed for Sequest™ searching. Positive protein identification was accepted for a peptide with Xcorr of greater than or equal to 3.50 for triply charged ions and 2.5 for doubly charged ions, and all with ΔCn ≥ 0.1, peptide probability ≤ 1e-3. The three peptides were identified as ubiquitin-like modifier activating enzyme 1(UBA1), isoform 1 of Fibrinogen alpha chain precursor and platelet factor 4(PF4), respectively (Table 3). Figures 3, 4 and 5 show the identification of specific b and y ions for sequences of the three peptides.

**Table 3:** Peptides sequencing and identification results

**Molecular weight**	**Amino acid sequence**	**International protein index**	**Peptide name**
3216.57 Da	HGFESGDFVSFSEVQGMVELNGNQPMEIK	IPI00641319	Ubiquitin-like modifier activating enzyme 1
4089.7 Da	GDSTFESKSYKMADEAGSEADHEGTHSTKRGHAKSRPV	IPI00021885	Isoform 1 of Fibrinogen alpha chain precursor
7762.87 Da	EAEEDGDLQCLCVKTTSQVRPRHITSLEVIKAGPHCPTAQLIATLKNGRKICLDLQAPLYKKIIKKLLES	IPI 00022446	Platelet factor 4

### Determination of serum PF4 by enzyme linked immunosorbent assay

**Figure 6:**
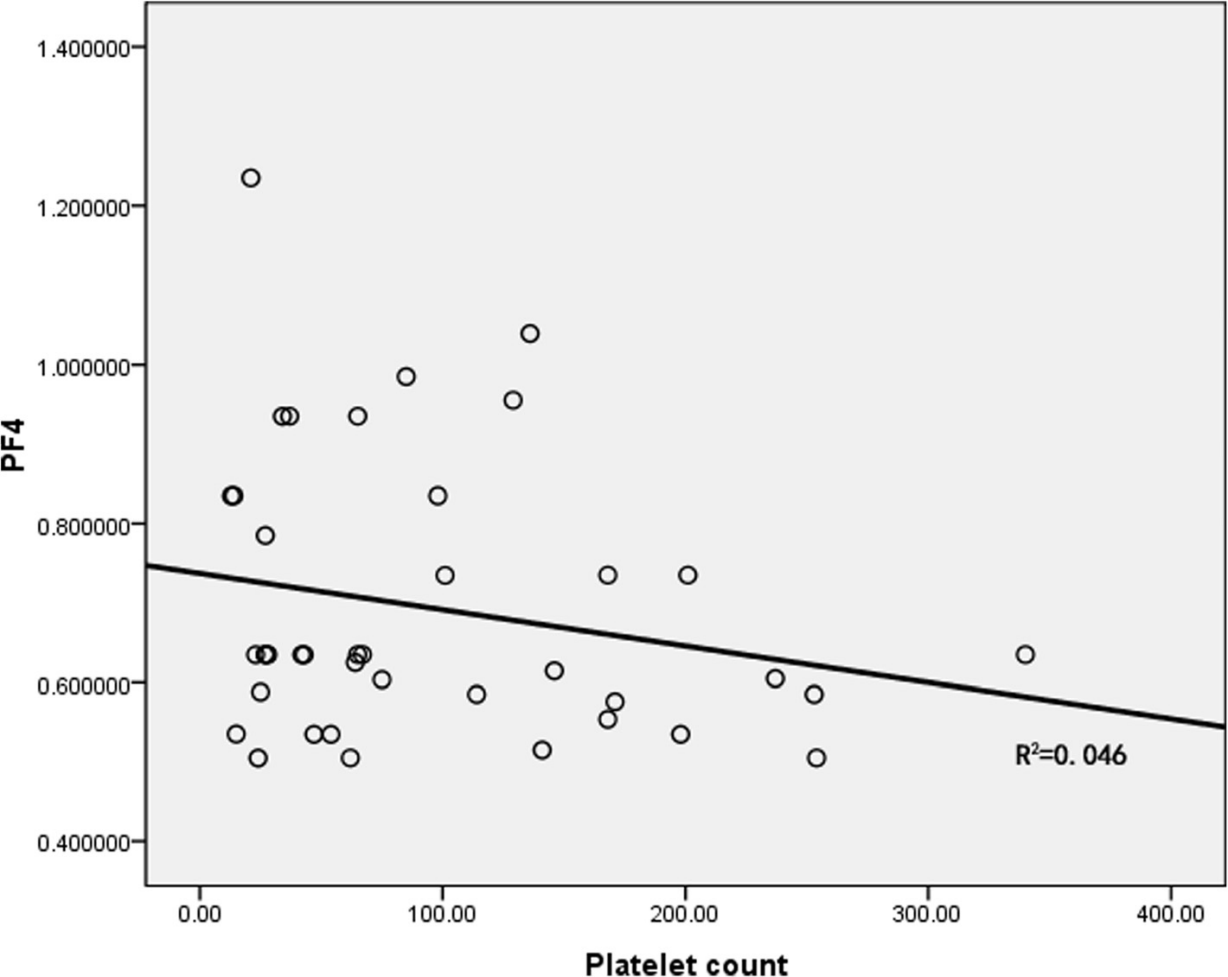
Correlation analysis between serum PF4 and platelet count in newly diagnosed AML.

We detected serum PF4 content by enzyme linked immunosorbent assay (ELISA) in 40 cases of newly diagnosed AML and 40 healthy controls. Compared with healthy control group, PF4 content in newly diagnosed AML group was decreased (newly diagnosed AML vs. control, 0.8012 ± 0.1876 vs. 3.2604 ± 1.0454, p = 1.8638E-6). To eliminate the influence of platelet count on serum PF4 content, correlation analysis was further done between platelet count and PF4 content. Correlation coefficient was 0.213 (p = 0.186),and no correlation was found between them (Figure 6). It follows that reduction of serum PF4 content in newly diagnosed AML is not due to thrombocytopenia.

### Validation of protein fragment by immunoblotting

**Figure 7:**
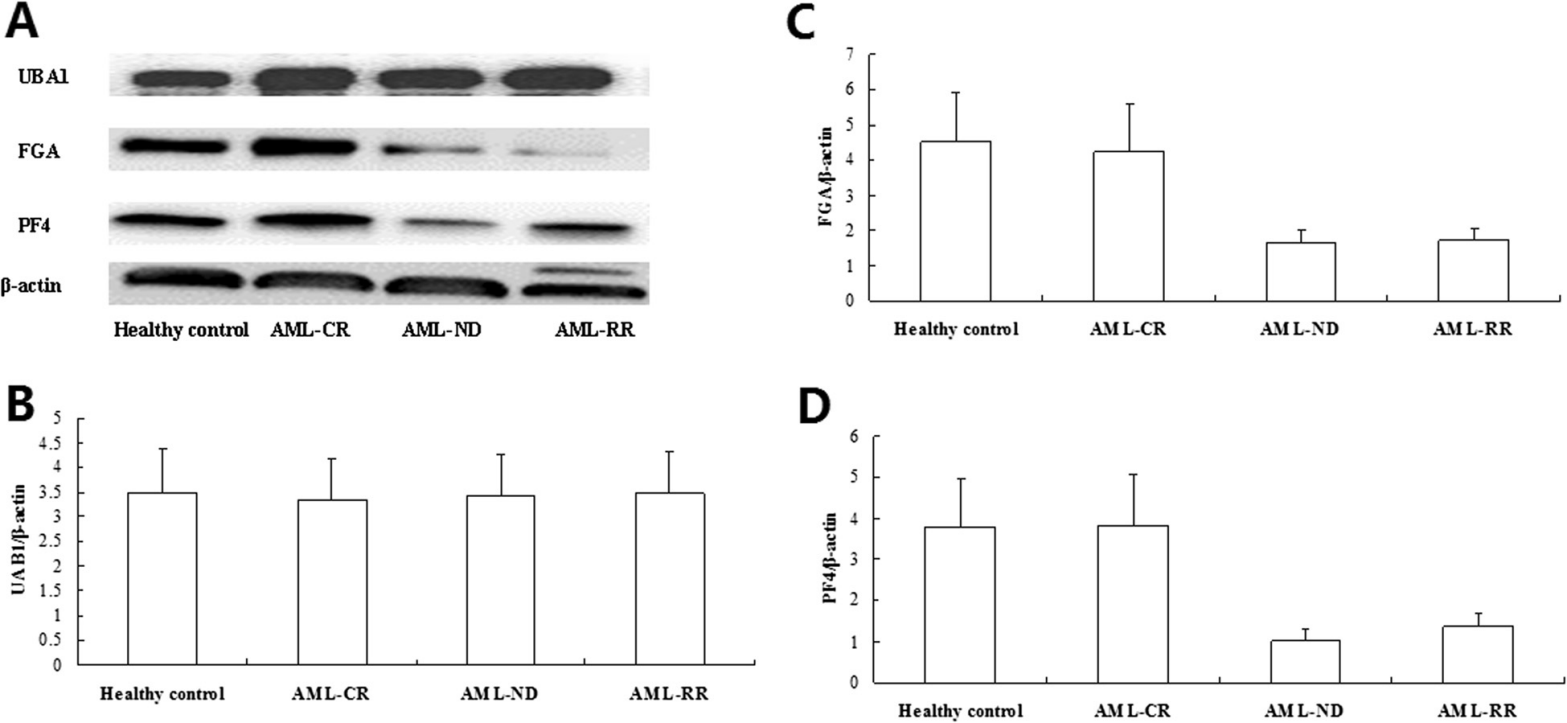
**Validation of protein fragment by immunoblotting. (A)** Levels of the UBA1 protein did not differ among the leukemia and normal cells. Isoform 1 of fibrinogen alpha chain precursor and PF4 immunoreactive bands show that weak or no bands are seen in newly diagnosed and refractory & relapsed AML cases. **(B)** Densitometry comparison of UBA1 protein relative to β-actin as determined by western blot analysis in figure A. **(C)** Densitometry comparison of isoform 1 of fibrinogen alpha chain precursor protein relative to β-actin as determined by western blot analysis in figure A. **(D)** Densitometry comparison of PF4 protein relative to β-actin as determined by western blot analysis in figure A. (UBA1: ubiquitin-like modifier activating enzyme 1; FGA: isoform 1 of fibrinogen alpha chain precursor; PF4: platelet factor 4; AML-CR: AML complete remission; AML-ND: AML newly diagnosed; AML-RR: AML refractory & relapsed).

Next, we sought to determine the level of UBA1, isoform 1 of fibrinogen alpha chain precursor and PF4 protein in AML and control samples by western blot analysis. Levels of the UBA1 protein did not differ among the leukemia and normal cells by immunoblotting (p = 0.849, Figure 7A,B). Isoform 1 of fibrinogen alpha chain precursor and PF4 immunoreactive bands show that weak or no bands are seen in newly diagnosed and refractory & relapsed AML cases (Figure 7A). Quantification of bands from western blot analysis showed decreased level of the two proteins in AML samples, relative to age-matched CR AML and healthy control samples significantly (p = 0.00368, p = 0.00067, Figure 7C,D).

### The relative intensity of three peptides in QC model in AML different groups

To explore the clinical significance of QC model, we compared the differences in the expression intensity of the three peptides (3216.57 Da, 4089.7 Da and 7762.87 Da) between AML newly diagnosed group, CR group, relapsed & refractory group and healthy control group (Table 4).

**Table 4:** Relative intensity of three peptides in AML different groups and healthy control group

**Mass**	**Ave1 ± StdDev1**	**Ave2 ± StdDev2**	**Ave3 ± StdDev3**	**Ave4 ± StdDev4**	**n1**	**n2**	**n3**	**n4**
3216.57	2.87 ± 1.45	1.83 ± 0.53	1.76 ± 0.48	3.08 ± 1.58	72	72	37	30
4089.7	2.69 ± 1.39	10.08 ± 4.03	11.63 ± 4.51	2.51 ± 1.35				
7762.87	1.94 ± 1.25	8.36 ± 3.35	8.62 ± 3.55	1.73 ± 1.18				

*Mass* mass to charge ratio value, *Ave* average peak intensity, *StdDev* standard deviation of the peak intensity, *n* number of patients, *1* AML newly diagnosed group, *2* healthy control group, *3* AML CR group, *4* AML refractory & relapsed group.

The relative intensity of peptide with MW of 3216.57 Da was elevated in the AML newly diagnosed group and the relapsed & refractory group, but no significant difference was observed between the two groups (p = 0.693). The relative intensity was reduced in the healthy control group and the CR group, no significant difference was observed between the two groups (p = 0.83) either. But the relative intensity was significant elevated in newly diagnosed group and refractory & relapsed group, compared with healthy control group and CR group (p = 0.00357, p =0.0012, p = 0.0027, p = 0.0073).

The relative intensities of the peptides with MW of 4089.7 Da and 7762.87 Da were both reduced in the AML newly diagnosed group and the refractory & relapsed group, but no statistical difference was observed between the two groups (p = 1.63, p = 0.53). The relative intensities were elevated in the healthy control group and the CR group, no significant differences were observed between the two groups (p = 1.92, p = 0.83) either, but the relative intensities were significantly reduced in the newly diagnosed group and the refractory & relapsed group, compared with healthy control group and CR group (p = 0.00357, p = 0.0012, p =0.0027, p = 0.0073, p = 0.00000108, p = 0.00000118, p = 0.00000001, p = 0.00000021).

### Impact of the relative intensity of three peptides on survival

**Figure 8:**
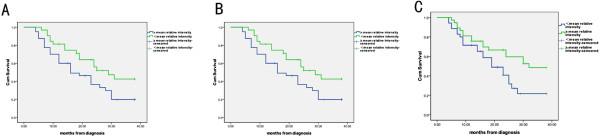
**Correlation between relative intensity of three peptides and overall survival (OS). (A)** Patients who had higher relative intensity of ubiquitin-like modifier activating enzyme 1(≥mean relative intensity) had a significantly inferior outcome. **(B)** The OS rate was higher in patients with increased relative intensity (≥mean relative intensity) of isoform 1 of fibrinogen alpha chain precursor. **(C)** Lower relative intensity of platelet factor 4(<mean relative intensity) associated with an unfavorable OS.

The median follow-up duration among 72 patients was 19 months (range 4–38 months). The patients were categorized into two groups according to the relative intensity of peptides (≥mean versus < mean). Kaplan–Meier analyses of overall survival (OS) showed that patients with higher relative intensity of ubiquitin-like modifier activating enzyme 1(≥mean relative intensity) had a significantly inferior outcome. Lower relative intensity of ubiquitin-like modifier activating enzyme 1(<mean relative intensity) was associated with an favorable OS (19.9 ± 7.0% versus 42.5 ± 9.8%, P = 0.037; Figure 8A). The OS rate was higher in patients with increased relative intensity (≥mean relative intensity) of isoform 1 of fibrinogen alpha chain precursor and platelet factor 4 than in those with decreased relative intensity (<mean relative intensity) (48.5 ± 10.7% versus 19.2 ± 7.2%, p = 0.015; 48.5 ± 9.0% versus 21.7 ±8.5%, P = 0.031, Figure 8B and C).

## Discussion

AML is a highly heterogeneous disease with distinct manifestations and prognostic and therapeutic implications. Leukemia MRD level monitoring contributes to impending relapse anticipation and clinical outcomes assessment, further guide’s clinicians to develop reasonable and effective treatment options so that patients can avoid unnecessary chemical drug toxicity.

Our QC model composed of three peptides achieved high sensitivity (90%) and specificity (93.33%) between newly diagnosed AML group and healthy controls. The peptides may be unique to AML and the AML specimen volume is up for augmentation for verification. The peptides could be purified to analyze their functions, so as to clear whether they are implicated in leukemogenesis.

Through document retrieval, there are only two workers applied ClinProt technology for studying AL patients serum peptide fingerprint to seek for the AL biomarkers. Elssner’s group made a comparative analysis of the serum peptide spectrum between childhood ALL and healthy controls and obtained an optimum discriminatory pattern proposed by ClinProTools after MB-HIC C8 enrichment. This model contained four relevant peaks: 2438 m/z, 6458 m/z, 7771 m/z and 9421 m/z. They may be four potential biomarkers for childhood ALL [21]. Liang’s group performed Copper-chelated magnetic bead fractionation/MALDI-TOF-MS analysis on sera from patients with newly diagnosed AML, CR after chemotherapy and healthy control, found that two peptides with m/z 1778.05 and 1865.13 were gradually decreased in their relative intensities with increase of remission degree, especially in molecular remission. With FT-ICR-MS detection, both peptides were identified as fragments of complement C3f, suggesting that the two peptides have the potential for monitoring of MRD [22].

The AML candidate peptides screened in our study are different from those in previous studies. The specific reasons are as follows: 1) Technically, we applied MB-WCX based ClinProt system. Some researchers made a comparison among beads with hydrophobic interaction chromatography C8 (MB-HIC-C8), MB-WCX and immobilized metal-ion affinity chromatography (MB-IMAC-Cu), indicating that the serum samples purified by MB-WCX group provided the best proteomic pattern. These samples had the most average peak numbers, the highest peak intensities, and the best capturing ability of low abundance proteins or peptides [23]. Besides low degree of automation, time-consuming and poor repeatability, traditional 2-DE had an ineffective detection of proteins with low-abundance, hydrophobicity, extreme alkalinity, extreme acidity, very high molecular weight (>200 kD) or very small molecular weight (<10 kD). The reproducibility of mass-spectral peaks abtained by protein chip-SELDI-TOF-MS in different research groups was very low. Target protein bound to the chip is difficult to elute for further identification. Label-free quantification strategies using high-resolution mass spectrometers do not require isotope label as internal standards and are economic as well as high-throughput. However, label-free quantitative proteomic techniques is still not mature enough and has great dependence on the reproducibility of the MS Experiment. Compared with isotope-labeling methods, label-free experiments need to be more carefully controlled due to possible error caused by run-to-run variations in performance of LC and MS [24]. The cDNA Microarray is applied to find disease-related markers from mRNA levels, whereas protein is the practitioner of human life activities. Protein with abnormal regulation and modification are always involved in malignant processes of AML with normal chromosomes and genes. 2) Serum specimens were as our study object, excluding the impact of fibrinogen and anticoagulant. Unlike some previous report, sera were pre-treated with urea and CHAPS. We used non-denatured serum samples. 3) We observed the peptides ranging from 700 to 10000 Da. Peptides as potential biological information molecules were quite abundant in serum. Comparing with proteins, peptides have the advantages of small MW, easy-transformed structure and easier chemical synthesis. Leukemogenesis is a complex process in which lots of peptides are involved. It needs high throughput proteomics technology to identify theses peptides. The structure of these peptides, their function and role in the pathological process of disease are expected to be further clarified so as to better defining disease pathogenesis and guiding clinical practice.

The relative intensity differences of the peptides in the QC diagnostic model were further compared in the AML different groups and the healthy control group. We found that the peptide with elevated relative intensity in newly diagnosed group was reduced after complete remission, when relapsed, its relative intensity again increased; the peptides which were down-regulated in the newly diagnosed group were upregulated after complete remission and down-regulated when relapsed. Kaplan–Meier analyses of OS show a significantly inferior outcome for patients with higher relative intensity of ubiquitin-like modifier activating enzyme 1 peptide fragment compared with the patients with lower relative intensity. The other two peptides with lower relative intensities were negatively correlated with patients’ overall survival. All these data suggest that these peptides can be used as potential predictive markers of treatment response, disease relapse, monitoring minimal residual disease and clinical outcome assessment.

Ubiquitin-like modifier activating enzyme 1 is also known as Ubiquitin-activating enzyme E1. Ubiquitin-proteasome pathway, an important protein quality control system in eukaryotic cells, is the major route by which cells rid themselves of excess proteins, such as damaged or unwanted intracellular proteins to regulate cell proliferation and differentiation, cell cycle progression, cell signal transduction, ultimately to maintain the cell normal function. Ubiquitination is a multistep enzymatic cascade in which ubiquitin is conjugated to target proteins with the assistance of E1 (Ubiquitin-activating enzyme), E2 (Ubiquitin-conjungating enzyme) and E3 (Ubiquitin ligase), then recognized, unfolded, and degraded by the proteasome enzyme complex [25,26].

The ubiquitin pathway has been implicated in several forms of malignancy through the degradation of tumor suppressor proteins [26]. In uterine cervical carcinomas, the tumor-suppressor protein p53 is tagged by the human papilloma virus (HPV) oncoprotein E6 for ubiquitin-mediated degradation resulting in transformed cells [27]. By immunoblotting, Xu’s group found the levels of ubiquitinated proteins were significantly increased in leukemia cell line K562, U937, NB4, THP1 and leukemia cells from 12 de novo AML patients compared with normal hematopoietic cells. But the levels of E1 protein in malignant cells did not differ from that in normal cells. Thus, these results suggest that the increased activity of pathway is not due to a greater abundance of the E1 enzyme, while E1 enzyme is more actively used in malignant cells. Inhibition of E1 enzyme activity of the four leukemia cell lines by RNA interference and chemical inhibitors PYZD-4409 can induce cell death, and an increase in p53 content, the same result was seen in multiple myeloma LP1 cell lines and the My-5 cells. Finally, in a mouse model of leukemia established by hMDAY-D2, Intraperitoneal administration of PYZD-4409 decreased tumor weight and volume compared with control without untoward toxicity [28]. We found that the relative intensity of serum ubiquitin-like modifier activating enzyme 1 peptide was elevated in newly diagnosed AML patients compared with healthy controls. But UBA1 protein levels in leukemia cells and normal cells are not different by immunoblotting. Maybe some growth negative regulator proteins of AML have an increased ubiquitination due to increased E1 enzyme activity but not a greater abundance of the E1 enzyme. These proteins are further degraded leading to leukemogenesis. The relative intensity of this peptide was declined in the AML patients in CR after chemotherapy and negatively regulate proteins might have a reduced degradation, but its relative intensity was increased again when AML relapsed, suggesting that the peptide may play a role in the pathogenesis of AML. It can be potential indicators for predicting AML treatment response and monitoring MRD. Its function and biological activity need further studies.

Fibrinogen is a glycoprotein composed of three pairs of unequal polypeptide chain. Fibrinogen alpha chain is just one of the three chains present in the circulating fibrinogen molecule (the other two being beta and gamma). A number of studies has shown that there are an increased expression of fibrinogen in many malignant tumors, such as pancreatic cancer, renal cell carcinoma, endometrial carcinoma, osteosarcoma, esophageal squamous cell carcinoma and so on [29]. Preston’s group considered that fibrinogen as an acute phase protein in pancreatic cancer had increased expression, because the result of increased synthesis of fibrinogen was associated with an ongoing inflammatory response to tumor [30]. Some studies suggest that tumor cells could promote coagulation process by interacting with endothelial cells and platelets, then by releasing active biological substances that activate platelets, which leads to high level of fibrinogen in the cancer patients’ blood. It is further indicated that fibrinogen could be an independent prognostic factor for disease-free, distant-free and overall survival in endometrial cancer, esophageal cancer and renal cell carcinoma [31-34]. The relative intensity of isoform 1 of fibrinogen alpha chain precursor peptide fragment was decreased in newly diagnosed AML and increased to normal level when patients received complete remission. When AML patients relapsed, the relative intensity was reduced again. The results have been validated by western blot analysis. We found fibrinogen α chain was highly expressed in newly diagnosed AML serum by ELISA (Data isn’t shown). It appears that a large part of the human serum peptidome as detected by MALDI-TOF MS is produced ex vivo by degradation of endogenous substrates by endogenous proteases. Peptides are generated during the proteolytic cascades that occur in the intrinsic pathway of coagulation and complement activation. Some of these are known bioactive molecules, others represent cleaved propeptides, and still others are seemingly random internal fragments of the precursor proteins. However, the observed cleavage sites are generally consistent with trypsin- and chymotrypsin-like activities of known serine proteases (kallikreins, plasmin, thrombin, factor I, etc.) [17]. The peptide identified (G592-V629) could be a putative plasmin- generated proteolytic fragments. In the light of this evidence, a down-regulation of the identified peptide (4089.7 Da) could be due to a hypothetical reduced enzymatic activity able to attack the C-terminal end of fibrinogen alpha chain.

PF4 is ELR (−) CXC chemokines, synthesized by megakaryocyte, stored in platelet alpha particles. Shi’s group used WCX2-Proteinchip arrays and SELDI-TOF-MS, HPLC-LC-MS/MS found that pediatric ALL patients had a reduced PF4 expression [35]. Kim’s group found that PF4 protein levels were a good indicator for the recovery of blood count in the complete remission of acute myeloid leukemia [36]. Our study showed that compared with healthy control group, the relative intensity of PF4 was reduced in newly diagnosed AML group. The relative intensity was elevated to normal level after AML patients achieved CR. When AML refractory & relapsed, the relative intensity was decreased again. By immunoblotting, PF4 protein was significantly decreased in newly diagnosed and refractory & relapsed AML cells. These findings are consistent with Kim’s. As PF4 is secreted by platelets and hematologic malignancies always have reduced platelets. In order to determine whether the reduced serum PF4 expression was associated with reduced platelet in MDS patients and benzene-exposed workers, studies revealed that lower PF4 expression was not due to thrombocytopenia [37,38]. We determined and compared serum PF4 content between newly diagnosed AML with and without thrombocytopenia. We found no difference between the two groups. Linear regression analysis showed that no correlation between PF4 content and platelet count.

## Conclusions

In conclusion, AML QC model had high sensitivity and specificity to discriminate AML patients from healthy controls by application of ClinProt system with high throughput and good repeatability. Ubiquitin-like modifier activating enzyme 1 peptide fragment, which is up-regulated in newly diagnosed AML patients, may decrease and approach to the normal level in their relative intensities after complete remission. When AML refractory & relapsed, the relative intensity is elevated again. Results are contrary to isoform 1 of fibrinogen alpha of chain precursor peptide fragment and PF4. Kaplan–Meier analyses of overall survival show that relative intensity of peptides is correlated with patient’s clinical outcome. We speculate these peptides can be used as potential markers for predicting AML relapse, monitoring minimal residual disease and assessing clinical outcome.

## Methods

### Patients’ clinical data and serum samples

This study was approved by the institutional Ethics Committee of the Hospital. 72 adult AML patients were randomly selected from those whose diagnosis was confirmed by myelocytic cytological diagnosis in the Second Affiliated Hospital of Xi’an Jiao Tong University during a given period of time (2009.1–2012.1). The diagnosis to adult AML was conducted based on the French-American-British (FAB) classification system. At the same time, 72 healthy controls were selected from adult healthy examinees without any diseases. In addition, 37 serum samples were obtained from AML patients with hematologic CR and 30 serum samples from refractory & relapsed AML patients. A day 15 bone marrow puncture were performed. The diagnostic standards of hematologic CR were: bone marrow blasts < 5%; absence of blasts with Auer rods; absence of extramedullary disease; absolute neutrophil count > 1.0 × 10^9^/L, platelet counts > 100 × 10^9^/L, independence of red cell transfusions. Resistant disease (RD) was defined as failure to achieve CR or CR with incomplete blood recovery (CRi ) (general practice; phase 2/3 trials), or failure to achieve CR, CRi, or partial remission(PR) (phase 1 trials); only includes patients surviving 7 days following completion of initial treatment, with evidence of persistent leukemia by blood and/or bone marrow examination. Relapse was defined as bone marrow blasts > 5%; or reappearance of blasts in the blood; or development of extramedullary disease. All patients and healthy examinees signed informed consents.

Clinical characteristics of newly diagnosed AML patients, patients with AML-CR and AML-RD are shown in Table 5. The serum samples were collected according to standard protocol. Fasting blood samples were collected from patients in the morning and allowed to clot at room temperature for 2 h. Sera were then separated by centrifugation at 2500 rpm for 10 min and stored at −80°C until analysis. The length of cryo-preservation period for all serum samples were less than 6 months[39]. For the reproducibility experiments, serum from each AML patient and each healthy control used the same MALDI-TOF-MS instrument to run three within-run assays and three between-run assays.

**Table 5:** Clinical features of patients in different AML groups before chemotherapy

**Clinical features**		**AML**					
		**Newly diagnosed**		**CR**		**Refractory & relapsed**	
Sex	male	38		19		13	
	female	34		18		17	
Age(year)		46(18–79)		48(18–69)		47(19–77)	
WBC(×10^9^/L)		23.5(0.25-143)		11.83(2.96-101.69)		23.58(0.94-123.6)	
Hb(g/L)		73(28–128)		111.5(69–135)		69(55–152)	
PLT(×10^9^/L)		26(12–287)		74(48–174)		31(9–172)	
Subtype		M0	0	M0	0	M0	0
		M1	6	M1	2	M1	3
		M2	8	M2	5	M2	2
		M3	6	M3	5	M3	0
		M4	24	M4	11	M4	13
		M5	28	M5	14	M5	12
		M6	0	M6	0	M6	0
		M7	0	M7	0	M7	0
Chromosome Abnormality		t(15;17)	6	t(15;17)	5	t(8;21)	1
		t(8;21)	4	t(8;21);	2	t(3;12)	2
		t(3;12)	2	11q23	2	11q23	4
		11q23	6			+8	3
		+8	4			−7	2
		−7	2				
Molecular Genetics Abnormality		PML-RARa	6	PML-RARa	5	AML-ETO	1
		AML-ETO	4	AML-ETO	2	MLL	3
		MLL	6			FLT3-ITD	5
		FLT3-ITD	6			C-kit Mut	2
		C-kit	3			NPM1 Mut	3
		NPM1 Mut	3				
Sternal tenderness		57/72		25/37		26/30	
Lymphadenectasis		39/72		14/37		21/30	
Splenohepatomegalia		36/72		16/37		14/30	
Curative effect		32/72		37/37		17/30	

This table showed clinical features of AML patients in different groups at the time of diagnosis. MLL refers to MLL rearrangement. Mut is the abbreviation of mutation. Curative effect refers to achieved complete remission after 2 courses of standard chemotherapy.

### Weak cation exchange magnetic beads based serum peptides purification

Weak cation exchange magnetic beads kit was purchased from Bruker Daltonics Inc. (Billerica, MA · USA). All purifications were performed in a one-step procedure according to manufacturers’ instructions through a standard protocol (ClinProtTM, Bruker Daltonics).

1) 5 μl serum samples, 10 μl Binding Buffer and 10 μl MB-WCX were mixed by pipetting in a 200 μL Orcugen sample tube. 2) After 5 minutes standing, the tube was placed in a magnetic bead separator for 1 min. Then the supernatant was discarded carefully with a pipette. 3) Subsequently 100 μl Washing Buffer was added, the tube was moved to and fro in two adjacent holes of the magnet separator, and then placed in the magnet separator for 1 min so as to make magnetic beads adhere to the wall of the tube. Afterwards, the supernatant was discarded carefully using a pipette. 4) Washing process was repeated twice. After binding and washing, 5 μl Elution Buffer was added to the tube to elute the bound peptides from the magnetic beads, and then the tube was placed in the magnet separator for 2 min. After that, the supernatant was transferred into a 0.5 ml tube with 5 μl Stabilization Buffer. Binding, Washing, Elution, and Stabilization Buffer were all provided by Bruker Daltonics Inc. 5) Matrix preparation: matrix solution with α-Cyano-4- hydroxycinnamic acid (HCCA) of 0.3 g/l in ethanol: acetone 2:1 (prepared freshly every day).

### Data acquisition by MALDI-TOF mass spectrometry

1) At first, 1 μL eluted sample was spotted onto a MALDI-TOF AnchorChip (TM) target (600 μm anchor diameter), and air-dried at room temperature, then 1 μL matrix (0.3 mg/ml HCCA, 50% ACN, 2% TFA) was spotted onto MALDI-TOF AnchorChip. 2) Anchorchip target plate was placed into the Micro flex mass spectrometer (Bruker Daltonics). 3) After calibration of instrument by cilnplot standard, samples were detected, scan range was 0.7-l0KD. FlexControl2.2 software was applied to acquire data and peptide profiling was constituted by different mass to charge ratio.

### Data processing and statistical analysis

Original mass spectrums were normalized by Flexanalysis 3.0 software, including soomthing and substrate baseline. Where after, we selected statistical algorithm built-in Clinprotools2.2 software for statistical analysis and acquization of differently expressed peptides. ANOVA test or Wilcoxon rank sum test was used to analyse peptide peak intensity differences in each group. Statistically significant was defined as p < 0.05. GA, SNN and QC were applied to establish model to distinguish AML and healthy control. The patients were categorized into two groups according to the relative intensities of peptides (≥mean versus < mean). Overall Survival was estimated by the Kaplan-Meier method and compared using a log-rank test. The event was defined as the time from initial diagnosis to treatment-related death time.

### Identification of peptide biomarkers

We utilized a nano-liquid chromatography–electro spray ionization–tandem mass spectrometry (nano-LC/ESI–mass spectrometry/mass spectrometry) system consisting of an Aquity UPLC system (Waters Corporation, Milford, USA) and a LTQ Obitrap XL mass spectrometer (Thermo Fisher, waltham, MA,USA) equipped with a nano-ESI source(Michrom Bioresources, Auburn, USA) to perform peptide sequencing and peptide identification. The peptide solutions purified by magnetic beads, were loaded to a C18 trap column (nanoACQUITY) (180 μm × 20 mm × 5 μm (symmetry)). The flow rate was 15 μl/min. Then the desalted peptides were analyzed by C18 analytical column (nano ACQUITY) (75 μm × 150 mm × 3.5 μm (symmetry)) at a flow rate of 400 nl/min for 60 min. The mobile phases A (5% acetonitrile, 0.1% formic acid) and B (95% acetonitrile, 0.1% formic acid) were used for analytical columns. The gradient elution profile was as follows: 5%B–45%B–80%B–80%B–5%B–5%B in 60 min. The spray voltage was 1.8 kV. MS scan time was 60 min. Experimental mode were Data Dependent and Dynamic Exclusion, scilicet a second cascade of the parent ion within 10 seconds added to the exclusion list for 90 seconds. Mass scan range was from m/z 400 to 2000. MS scan used Obitrap, resolution was set at 100000. CID and MS/MS scan applied LTQ. In MS spectrogram, we selected single isotope composed of 10 ions with strongest intensity as parent ion for MS/MS (Single charge being excluded, not as parent ion). We applied data analysis software Bioworks Browser 3.3.1 SP1 for Sequest™ retrieving. Retrieval Database was International Protein Index (IPI human v3.45 fasta with 71983 entries). Parent ion error was set at 100 ppm, fragment ions error at 1 Da, digested mode at non-digested and variable modifications at methionine oxidation.

### Determination of serum PF4 by ELISA

We used ELISA to assay serum PF4 content in 40 newly diagnosed AML patients and 40 healthy controls and compared the differences between the two groups. Detailed procedure was according to manufacturers’ instructions of ELISA kit (R&D, USA). Furthermore, we analyzed the correlation of platelet counts and serum PF4 content in AML patients. Independent sample t-test comparative analysis was done through the statistical software SPSS 17.0. Correlation analysis used linear regression analysis. Statistically significant was defined as p < 0.05.

### Western blot analysis for validation

Sodium dodecyl sulfate polyacrylamide gel electrophoresis (SDS-PAGE) and immunoblotting were performed essentially as described elsewhere. Briefly, cell pellets were resuspended on ice in lysis buffer containing 10 mM Tris–HCl (pH 7.4), 5 mM MgCl2, 1% Triton X-100, 100 mM NaCl, 10 mM NaF, 1 mM Na3VO4 and a protease inhibitor cocktail. After sonication, cellular proteins were separated on an SDS-polyacrylamide gel and transferred to polyvinylidene fluoride membranes (Roche Diagnostics Corporation, Indianapolis, Indiana United States), which were probed with the appropriate primary antibodies. Immunoreactivity was detected with the relevant horseradish peroxidase-labeled secondary anti-bodies which, in turn, were visualized on an Image Reader Tano-5500 (Tano, Shanghai, China) using chemiluminescence substrate reagent purchased from 7 sea pharmtech (Shanghai, China) For quantification of the data, the images were further analyzed on the same instrument using 2D Densitometry Image Analyzer IPP 7.0 software (Tano, Shanghai, China).

## Abbreviations

MRD: Minimal residual disease; MB-WCX: Weak cation exchange magnetic beads; MALDI-TOF-MS: Matrix assisted laser desorption ionization time of flight mass spectrometry; AML: Acute myeloid leukemia; HPLC-MS/MS: High-performance liquid chromatography tandem mass spectrometry/mass spectrometry; CR: Complete remission; QC: Quick classifier; UBA1: Ubiquitin-like modifier activating enzyme 1; PF4: Platelet factor 4; RTQ-PCR: Real-time quantitative polymerase chain reaction; SELDI-TOF-MS: Surface enhanced laser desorption ionization time of flight mass spectrometry; 2-DE: Two-dimensional gel electrophoresis, Rho-GDP; MM: Multiple myeloma; SNN: Supervised neural network; CV: Coefficient of variation; MW: Molecular weight; GA: Genetic algorithm; ELISA: Enzyme linked immunosorbent assay; OS: Overall survival; HPV: Human papilloma virus.

## Competing interests

The authors declare no conflict of interests with any company or financial organization.

## Authors’ contributions

JB and YZ were involved in serum peptide purification, writing the draft of the manuscript; JY performed serum peptide profiling data acquisition and processing; YY and JLW were involved in serum samples and clinical data collection; ALH, WGZ and CH designed the experiments and supervised the research manuscript. All authors read and approved the manuscript.

## Supplementary Material

Additional file 1: Figure S1 Serum peptide fingerprints of the same serum through same processing in three-repeated experiments. **(A)** Serum peptide fingerprints of the same acute leukemia through same processing in three-repeated experiments. **(B)** Serum peptide fingerprints of the same healthy control through same processing in three-repeated experiments. Click here for file
