# Supplementary figures and images for: Potential biomarkers for adult acute myeloid leukemia minimal residual disease assessment searched by serum peptidome profiling
Source: Proteome Sci. 2013 Aug 3;11:39. doi: 10.1186/1477-5956-11-39 (PMC3751134; doi:10.1186/1477-5956-11-39)

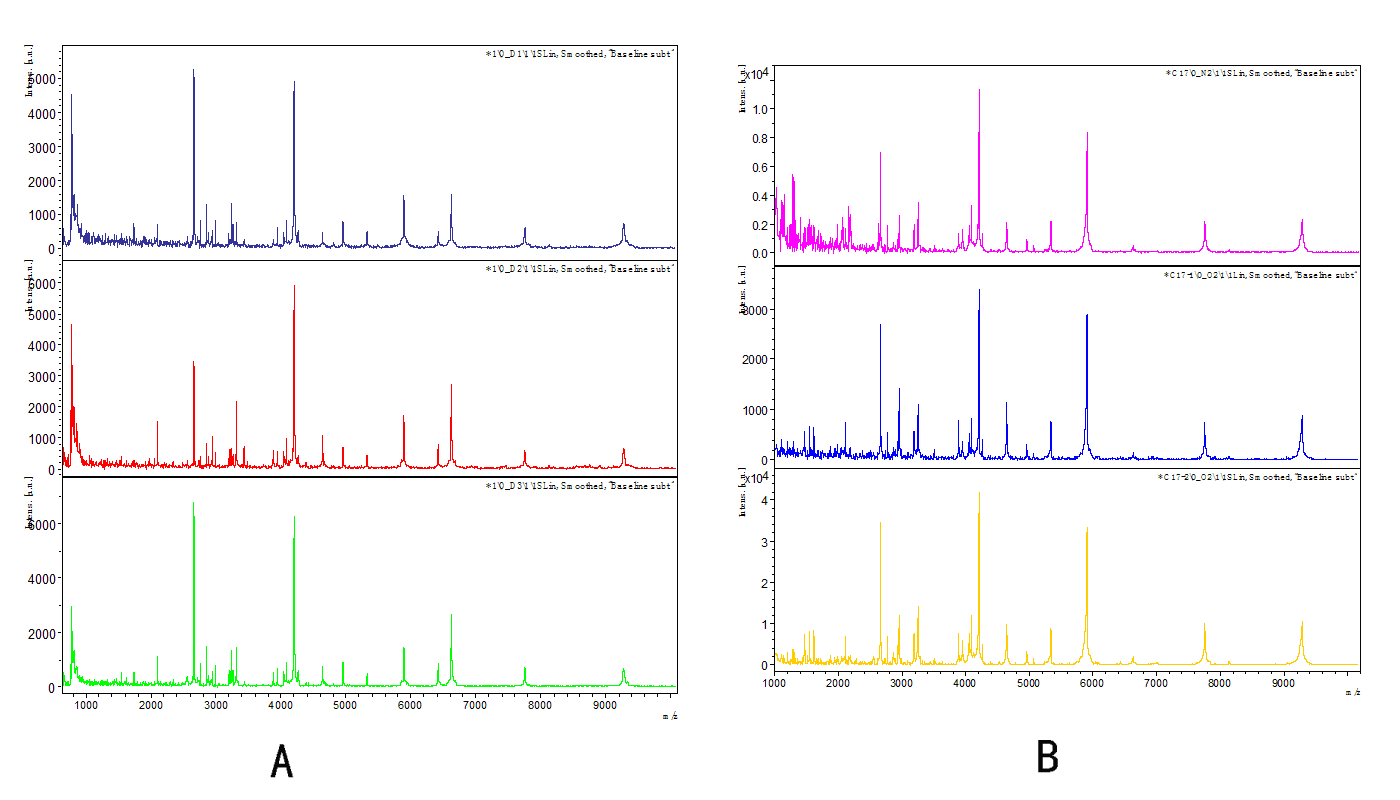

Supplement: Additional file 1: Figure S1 — Serum peptide fingerprints of the same serum through same processing in three-repeated experiments. (A) Serum peptide fingerprints of the same acute leukemia through same processing in three-repeated experiments. (B) Serum peptide fingerprints of the same healthy control through same processing in three-repeated experiments. [file 1477-5956-11-39-S1.tiff]
